# Translation of a scale measuring cognitive test anxiety (G-CTAS) and its psychometric examination among medical students in Germany 

**DOI:** 10.3205/zma001343

**Published:** 2020-09-15

**Authors:** Alexandra Stefan, Christina M. Berchtold, Matthias Angstwurm

**Affiliations:** 1 Ludwig-Maximilians-Universität München, Zentrale PJ-Koordination, München, Germany; 2 Ludwig-Maximilians-Universität München, LMU-StaR (Staatsexamensrepetitorium), München, Germany; 3 Ludwig-Maximilians-Universität München, Klinikum der Universität München, Medizinische Klinik und Poliklinik IV, München, Germany

**Keywords:** test anxiety, cognitive test anxiety scale, worry, item analysis, validity check, test performance, gender, medical students, state examination, psychological stress

## Abstract

**Aim of the study: **Test anxiety expresses itself in a variety of physical and cognitive processes. Due to its influence on test performance, the cognitive component in particular can have a negative impact on those affected. A measuring instrument for this is not yet available in the German-speaking world but does exist in the form of the “Cognitive Test Anxiety Scale” (CTAS), among other languages, in English. The aim of this work was the creation and psychometric review of a German version of the scale (G-CTAS).

**Methods: **A German translation of the scale was created using a forward-backward procedure. Statistical investigations were then carried out on a cohort of medical students, which included an item analysis with calculation of difficulty, variance and item discrimination as well as the determination of the scale’s internal consistency. The criterion validity was examined using test performance and gender-specific differences.

**Results: **The final version contains 26 matching items with acceptable item parameters (mean values >1.46, <3.13; variances >0.48; part-whole-discrimination-indices >0.37). Cronbach's alpha was 0.92, the scale was therefore found to be a reliable measuring instrument. The scale validity could be confirmed by significant differences (p<0.01) between total values of female and male participants as well as significant correlations (p<0.001) between total values and test performance in the written and oral part of the first state examination.

**Conclusion: **With G-CTAS a suitable measuring instrument for cognitive test anxiety within the German-speaking world is available, which can be used, among other things, for studies concerning the relationship between stress, exams and test anxiety among medical students.

## Zusammenfassung

**Zielsetzung:** Prüfungsangst äußert sich in einer Vielzahl körperlicher und kognitiver Prozesse. Durch ihren Einfluss auf Prüfungsleistungen kann sich besonders die kognitive Prüfungsangstkomponente negativ auf Betroffene auswirken. Ein Messinstrument hierfür ist im deutschen Sprachraum bisher nicht verfügbar, existiert aber in Form der „Cognitive Test Anxiety Scale“ (CTAS) unter anderem in englischer Sprache. Ziel dieser Arbeit war die Erstellung und psychometrische Überprüfung einer deutschen Version der Skala (G-CTAS).

**Methodik: **Mithilfe eines Vorwärts-Rückwärts-Verfahrens wurde eine deutsche Übersetzung der Skala erstellt. An einer Kohorte Humanmedizinstudierender wurden anschließend statistische Untersuchungen durchgeführt, welche eine Itemanalyse mit Berechnung von Schwierigkeit, Varianz und Trennschärfe sowie die Ermittlung der internen Skalenkonsistenz beinhalteten. Eine Überprüfung der Kriteriumsvalidität erfolgte über Prüfungsleistungen sowie geschlechtsspezifische Unterschiede.

**Ergebnisse: **Die finale Version enthält 26 inhaltlich passende Items mit akzeptablen Itemkennwerten (Mittelwerte >1,46, <3,13; Varianzen >0,48; Trennschärfeindices >0,37). Cronbach’s alpha betrug 0,92, die Skala stellt somit ein reliables Messinstrument dar. Die Skalenvalidität konnte durch signifikante Unterschiede (p<0,01) zwischen Summenwerten weiblicher und männlicher Teilnehmer sowie signifikante Korrelationen (p<0,001) zwischen Summenwerten und Prüfungsleistungen im schriftlichen und mündlichen Physikum bestätigt werden.

**Schlussfolgerung: **Mit G-CTAS steht ein geeignetes Messinstrument kognitiver Prüfungsangst auch für den deutschen Sprachraum zur Verfügung, welches sich unter anderem für Untersuchungen des Zusammenhangs zwischen Stress, Prüfungen und Prüfungsangst unter Medizinstudierenden anwenden lässt.

## Introduction

Test anxiety is counted among the anxiety disorders. According to the "Diagnostic and Statistical Manual of Mental Disorders, 5^th^ Edition" (DSM-5 [1]) it is classified as social phobia and according to the “International Statistical Classification of Diseases and Related Health Problems, 10^th^ Revision” (ICD-10 [2]) encoded as a specific phobia [3]. There is no generally accepted and therefore binding definition of test anxiety, but a theory of two components is common. It differentiates the two main components “emotionality” and “worry” [4]. Physiological symptoms such as sweating, increased heart rate and nervousness are attributed to the “emotionality” component. The “worry” component, on the other hand, is attributed with a large number of cognitive processes, originally described by Liebert & Morris as “any cognitive expression of concern about one’s own performance” [4]. This includes i. a. comparing the own performance with comparison groups, thinking about the consequences of failing and showing low self-confidence as well as loss of self-esteem. Due to the great breadth and complexity of the processes that make up the "worry" component it is also known as cognitive test anxiety.

Test anxiety has multiple effects. A negative impact on the subjective well-being of those affected [5], [6], interrelationships with social and specific phobias [7] and a correlation with depressive symptoms [8] are known. Connections between increased test anxiety and reduced test performance were repeatedly observed [9], [10], [11], [12], [13], [14], [15]. These relationships were commonly noticed for the cognitive component, while the influence of the affective component was mostly rated as neutral or even positive [16], [17].

Various studies show that women report higher values for general and cognitive test anxiety than men [13], [15], [17], [18], [19], [20], a clear cause for this could not be identified so far.

According to Powell [21], medical students also represent a risk collective for test anxiety. Different studies have shown that aspiring doctors are a group of people with a high psychological burden [22], [23], [24]. Depression and anxiety increase at the start of their studies [25] while mental health decreases [26]. These developments are also relevant because they continue into the working life [27], [28] and can have a negative impact on patient safety through reduced treatment quality [29]. Exams are an issue that should not be underestimated in these developments as they are considered to be a relevant trigger for stress among students due to fear of being overworked and under pressure to perform [30]. 

Several questionnaires for measuring test anxiety have already been established and some have been translated into other languages. They are pursuing different approaches: Rost & Schermer's “Differential Anxiety Inventory” [31] records triggering and sustaining conditions and thus enables advice and therapy-oriented diagnostics. By contrast, the “Test Anxiety Inventory” by Spielberger [32] and its revised versions [33], [34], [35] are suitable for measuring the components described by Liebert & Morris. The “Cognitive Test Anxiety Scale” (CTAS) by Cassady & Johnson [36] was primarily developed for measuring and examining cognitive test anxiety. The scale and its revised versions have already been used for numerous studies abroad [37], [38], [39], [40], [41], [42], [43], [44], [45], [46] and have been translated into several languages [47], [48], [49], [50]. 

## Problem & objectives

Due to the various negative effects, a more thorough understanding of the causes of test anxiety and the identification of risk groups is important. In the long term, there is also further research needed to develop treatment approaches. This is especially true in the context of medical studies. Precondition for this is the exact measurement using a qualified and reviewed scale. Since existing German scales are not explicitly suitable for measuring cognitive test anxiety and so far no German version of CTAS is available, the possibilities in German-speaking countries are limited. Therefore, the aim of the study was to translate CTAS into German, to statistically examine the items and to investigate the reliability and validity of the scale.

## Material & methods

The study project was supervised by the ethics committee of the Ludwig-Maximilians-University of Munich (LMU) and declared as ethically unproblematic (application number 166-15).

### Sample

Between autumn 2015 and autumn 2016 an online survey was conducted among medical students of the LMU after the first section of the medical state examination (first state examination). After giving their consent, all students who had been in the 4^th^ or a higher pre-clinical semester at the time of the exam and who had therefore potentially taken part in the first state examination were contacted via e-mail via the university's mailing list. Answering the questionnaire was voluntary, there were no advantages for the students from participating in the study or disadvantages from not participating. Only fully processed questionnaires were included in the analysis. The sample consisted of a total of 291 students, the majority was female (female: n=191, 65.6%; male: n=100, 34.4%), the average age was 22.75 (SD 4.26). The sample size made it possible to carry out an item analysis and determine the internal consistency and criterion validity of the scale, but the size was not sufficient to carry out a factor analysis [51]. 

#### Questionnaire

For the online survey, a questionnaire was created consisting of demographic data, an instrument for measuring cognitive test anxiety and information on test performance.

##### Demographic data

Participants were initially asked about their age and gender.

##### Cognitive test anxiety

**Cognitive Test Anxiety Scale (CTAS)**

CTAS contains different facets of cognitive test anxiety, a wide range of symptoms are queried, including task-irrelevant thinking during the test preparation and during the test itself, the comparison with others, invasive thoughts during learning for the test and the examination itself as well as the tendency to skip relevant task details in examinations [36]. All 27 items from the original version of CTAS [36] were used in the study of which nine items are inverse-coded. The answer is given on a four-point Likert scale, which ranges from strong rejection to strong approval. Strong rejection is rated with one point, strong approval with four points. After recoding the inverse-coded items, high scale values are indicative of a high degree of cognitive test anxiety. The 27-point scale has a high internal consistency and high criterion validity [48]. It also proved to be a stable and consistent measure of cognitive test anxiety with high predictive power for test performance. In pilot studies a high degree of agreement was shown on the already established instruments “Test Anxiety Inventory” by Spielberger [32] and "Reactions to Tests" by Sarason [11], [36].

**Translation of CTAS into German**

All 27 items were translated using a forward-backward method. The authors created a first German translation with the focus less on a literal than on a content-oriented translation. The translation was discussed with a native English speaker and a preliminary version of the scale was created. This was then independently translated back into English by three bilingual persons. The preliminary version was compared with the back translations, deviations in content were identified and discussed. After all, a final version of the scale was created.

##### Test performance

Participants were asked about their test grades in the written and oral part of the first state examination, the specification of both grades was voluntary.

#### Analyses

##### Item analysis 

Initially the nine inverse-coded items were recoded and retained for all subsequent analyses. Descriptive statistics of the items, including mean (M), standard deviation (SD) and variance (V), were calculated. Items with a very high level of difficulty of less than 10% (M<1.3) or very low level of difficulty of over 90% (M> 3.7) were reviewed in terms of content and removed from the scale if the wording was inadequate. A review of the content also took place if an item showed a relatively small variance or the response format was not fully exhausted. Item discrimination indices were calculated using part-whole corrected discrimination indices [52]. Items with low discrimination indices (r_it_<0.3) were also reviewed for their content and removed if necessary. After an item was removed, discrimination indices of the remaining items were calculated again.

##### Scale reliability

To assess the reliability of the scale, its internal consistency was calculated using Cronbach’s alpha. The scale was considered reliable if Cronbach’s alpha >0.8 [53].

##### Criterion validity

To determine the criterion validity of the scale, total values between female and male participants were compared using a t-test. The relationship between scale total values and test performance in the written and oral part of the first state examination was examined using Pearson correlation coefficient (r_p_).

#### Programs

The “Statistical Package for the Social Sciences” (SPSS) 25 and Microsoft Excel 2013 were used for statistical data analysis.

## Results

### Item analysis

#### Item difficulty

All 27 items of the original English scale [36] are shown in table 1 (Tab. 1) together with descriptive statistics of their German version. The difficulty of the items ranged between M=1.46 (SD=0.74; item 20: *“When I take a test that is difficult, I feel defeated before I even start.”*) and M=3.13 (SD=0.77; item 13: *“I do well in speed tests in which there are time limits.”*) and therefore within the defined interval of difficulty. Variances of the items ranged from V=0.48 (item 19: *“During tests, I have the feeling that I am not doing well.”*) and V=1.11 (item 25:* “I feel under a lot of pressure to get good grades on tests.”*). Item 4 *(“I tend to freeze up on things like intelligence tests and final exams.”*), 19 (*“During tests, I have the feeling that I am not doing well.”) and 20 (“When I take a test that is difficult, I feel defeated before I even start.”*) showed the comparatively smallest variances, but, like the other items, made full use of the response format. Since there was also no indication of inadequate wording, all three items were retained.

##### Item discrimination

When calculating the discrimination indices, an insufficient fit from item 25 (“*I feel under a lot of pressure to get good grades on tests.”*) to the rest of the scale was noticed, which was confirmed after a review of the content (see table 2 (Tab. 2)). Item 25 had already been identified as insufficient by Furlan et al. [48] in terms of content and was subsequently removed from the scale for all consequent analyses. After the removal of item 25, the discrimination indices of the other items ranged between r_it_=0.37 (item 3:* “I have less difficulty than the average college student in getting test instructions straight.”*) and r_it_=0.68 (item 14: *“During a course examination, I get so nervous that I forget facts I really know.”*) and thus above the previously determined value.

#### Scale reliability

Cronbach’s alpha of the scale was 0.92.

#### Criterion validity

G-CTAS total values of the female participants averaged 59.76 (SD=12.67), G-CTAS total values of the male participants averaged 54.22 (SD=13.59). The values differed significantly from each other in the t-test (mean difference: 5.54, T=3.46, df: 289, p<0.01). G-CTAS total values correlated significantly (p<0.001) with grades in the written (r_p_=0.44) and oral part of the first state examination (r_p_=0.40).

## Discussion and conclusions

In the present study, a German version of CTAS was created and statistically reviewed and validated on a cohort of medical students. Analyses showed that 26 items of the German version have acceptable difficulty, variance and item discrimination. G-CTAS also has a high internal consistency and construct validity.

Due to its size and gender distribution, the sample is representative of a German medical faculty. These were medical students of similar ages in the same study section and thus a highly selected sample, which is why the results are less suitable for creating standard values or for defining severity of cognitive test anxiety. However, this qualifies the scale particularly for further studies on cognitive test anxiety among medical students. The item formulations enable the use of the scale for researches of cognitive test anxiety during study without any restriction regarding the subject. Further studies at German faculties can therefore follow without adapting the scale. 

Since a method effect could be observed repeatedly through the use of inverse-coded items, several revised versions of the scale already exist [42], [43], [44]. In the course of this work the translation of the original scale was chosen because the short form of the scale proposed by Cassady & Finch [42] can be easily generated by removing all inverse-coded items.

The items were not selected on the basis of a characteristic value, but in terms of difficulty, variance and item discrimination. The wording of the content played a decisive role in excluding an item. According to Bühner [51], the content should have highest priority when selecting items, but this method has rarely been used in previous analyzes with CTAS and its translations. Nevertheless, the result of the item analysis coincides with the observations by Furlan et al. [48], which also led to the exclusion of item 25. Item 25 probably measures primarily performance pressure and less cognitive test anxiety.

Cronbach’s alpha of G-CTAS was also comparable to preliminary investigations [48]. The value indicates a high internal consistency and thus for a homogeneous scale. A detailed analysis of the factor structure could not be conducted because the sample size was not sufficient for an exploratory and subsequent confirmatory factor analysis [51]. This should be carried out as the next step, since precise knowledge of the scale’s structure is not only the basis for the formation of standard values but could also contribute to a more thorough understanding of the causes and effects of test anxiety.

Differences between female and male participants, as well as significant, positive correlations with test performance were able to prove the criterion validity of the German scale. The study design did not address gender-specific differences in cognitive test anxiety, which is why their causes still remain unclear. In contrast, the study design made it possible to compare the connections between cognitive test anxiety with written and oral exams. Here, a significant correlation with both test modalities was observed at a similarly high level of correlation.

The connection of cognitive test anxiety with both, written and oral exams, once again illustrates the possible extent for those affected. Connections with the performance in a relevant test such as the first state examination emphasize how decisive test anxiety can be in relation to academic achievement. Finding the causes of test anxiety and treatment approaches is not only important from the perspective of those affected but should also be of high priority for universities. Medical students represent a risk collective regarding mental stress and test anxiety and should be supported in this regard. A more precise understanding of the dynamics between stress, exams and test anxiety is essential in order to identify people at risk and to be able to intervene in these negative developments at an early stage. The basis for this is a suitable and most accurate measuring instrument of cognitive test anxiety. With G-CTAS this is now also available in German-speaking countries.

## Acknowledgements

Our thanks go to Dr. Amanda Tufman, Mrs. Sarah Garcia, Mrs. Franziska Enders and Mrs. Miruh Lee for their assistance in translating the scale.

## Competing interests

The authors declare that they have no competing interests. 

## Figures and Tables

**Table 1:**
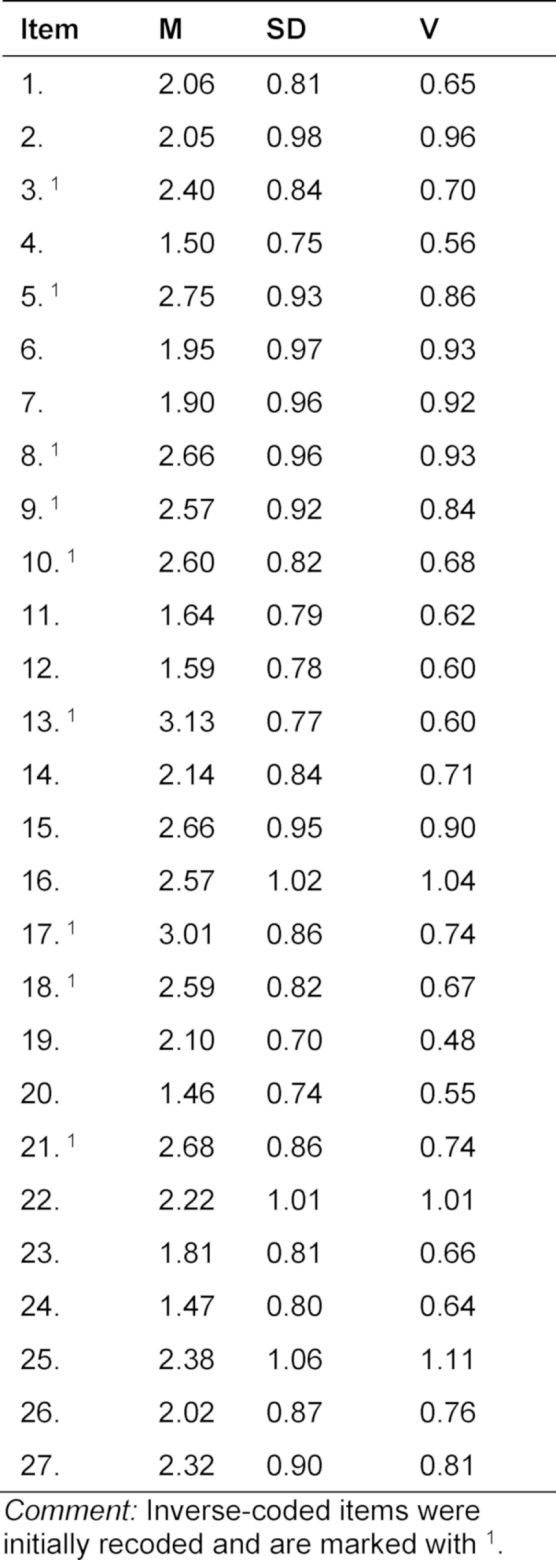
Mean (M), standard deviation (SD) and variance (V) of all 27 translated items are shown.

**Table 2:**
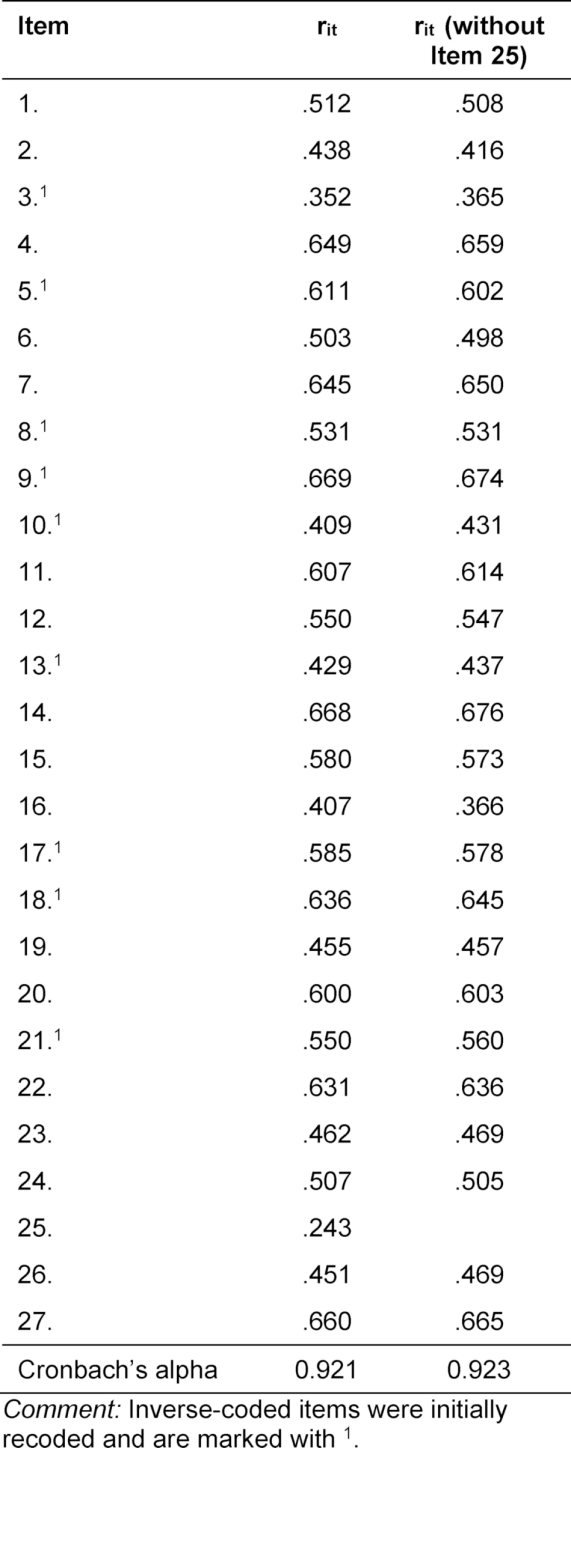
Discrimination indices of all 27 items [36] as well discrimination indices after removal of item 25 (r_it_ [without item 25]) are shown. Furthermore, Cronbach’s alpha is presented for both scales.
